# Suppression of Fatty Acid and Triglyceride Synthesis by the Flavonoid Orientin through Decrease of C/EBPδ Expression and Inhibition of PI3K/Akt-FOXO1 Signaling in Adipocytes

**DOI:** 10.3390/nu10020130

**Published:** 2018-01-26

**Authors:** Shiori Nagai, Chihiro Matsumoto, Makio Shibano, Ko Fujimori

**Affiliations:** 1 Department of Pathobiochemistry, Osaka University of Pharmaceutical Sciences, 4-20-1 Nasahara, Takatsuki, Osaka 569-1094, Japan; oups0645@gmail.com (S.N.); e17904@gap.oups.ac.jp (C.M.); 2 Department of Pharmacognosy, Osaka University of Pharmaceutical Sciences, 4-20-1 Nasahara, Takatsuki, Osaka 569-1094, Japan; shibano@gly.oups.ac.jp

**Keywords:** adipogenesis, orientin, C/EBPδ, PI3K/Akt, FOXO1

## Abstract

Plant flavonoids have a variety of biological properties. In a previous study, we found that the tea of the Asian dayflower, *Commelina communis* L., decreased the body weight gain in high-fat diet-fed mice. In this study, we studied the anti-adipogenic ability of a flavonoid orientin that is found in abundance in *C. communis*. Orientin repressed the accumulation of intracellular triglyceride (TG) in mouse adipocyte 3T3-L1 cells. The treatment with orientin also decreased the mRNA levels of the genes involved in adipogenesis, lipogenesis, lipolysis, and TG synthesis, and reduced the release of glycerol. Orientin lowered the expression of CCAAT/enhancer binding protein (C/EBP) δ in the early stage of adipogenesis, leading to a decrease in the expression of the adipogenic master transcription factors such as peroxisome proliferator-activated receptor (PPAR) γ and C/EBPα. Moreover, the anti-adipogenic effect of orientin repressed the phosphorylation of Akt and subsequent phosphorylation of forkhead box protein O1 (FOXO1), which inhibits the transcription of the *Ppar* gene. These results indicate that a plant flavonoid orientin suppressed the expression of the *Pparγ* gene through repression of *C/ebpδ* expression and inhibition of the phosphoinositide 3-kinase /Akt-FOXO1 signaling in adipocytes.

## 1. Introduction

Obesity is now a global health problem in developed and developing countries [1], and increases the incidence of metabolic diseases such as cardiovascular diseases and type II diabetes [1,2,3]. Obesity is thought to be attributed to a number of factors such as unhealthy eating habits, aging, and lack of exercise. Obesity is associated with increases in cell size and cell number of adipocytes, which are derived from an imbalance between energy intake and energy expenditure. Adipocytes are specialized cells that store energy as lipids and when needed, the stored lipids are hydrolyzed and oxidized to control energy balance in the body [4]. Moreover, fat tissue is now known as an endocrine organ that produces a variety of adipocytokines [5].

Adipocyte differentiation (adipogenesis) is controlled via complex processes, and a number of transcription factors involved in its regulation have been found [6]. Among them, three transcription factors, peroxisome proliferator-activated receptor (PPAR) γ, CCAAT/enhancer binding proteins (C/EBPs), and sterol regulatory element-binding protein-1, are important in the regulation of adipogenesis [6]. Moreover, C/EBPβ and C/EBPδ, which are the transcription factors acting in the early stage of adipogenesis, enhance the transcription of *C/ebpα* and *Pparγ* genes to activate the terminal differentiation of adipocytes [7]. Furthermore, once PPARγ and C/EBPα are activated, they enhance gene expression of each other and then induce the expression of various adipogenic genes that are needed for maintaining adipocyte characteristics and terminal differentiation.

**Figure 1:**
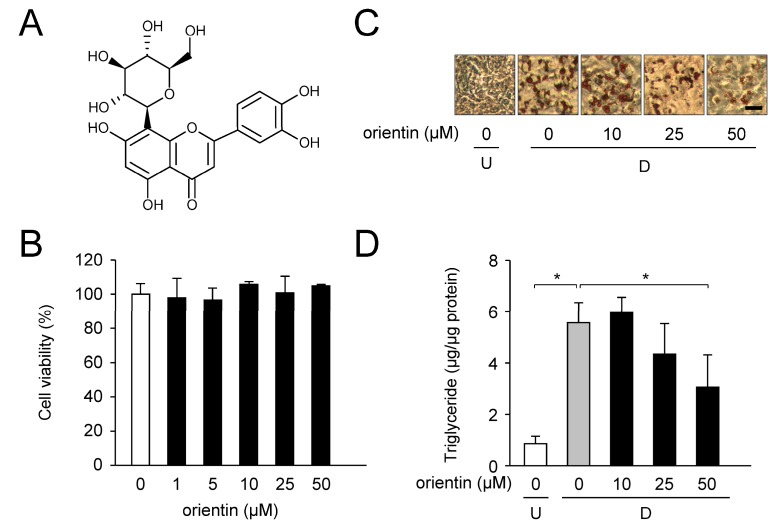
Decrease in accumulation of intracellular lipids by orientin. (**A**) Chemical structure of orientin; (**B**) Cytotoxicity of orientin in 3T3-L1 cells. Cell culture was performed for 6 days in medium including various concentrations of orientin (0–50 μM). Cytotoxicity was measured by WST assay. Data are the means ± SD from three experiments; (**C**) Oil Red O staining of the lipid droplets in 3T3-L1 cells. The undifferentiated cells (U) were differentiated into adipocytes (D; for 6 days in medium with orientin (0–50 μM). Bar = 50 μm; (**D**) Change in intracellular triglyceride (TG) level in 3T3-L1 cells. Undifferentiated cells (U; 0 μM orientin, white column) were caused to differentiate into adipocytes ((**D**) 0 μM orientin, gray column) for 6 days in medium containing orientin, (10, 25, or 50 μM; black columns). Data are shown as the means ± SD from three experiments. * *p* < 0.01, as shown by the brackets.

Flavonoids are natural polyphenolic compounds derived from fruits and vegetables [8]. There are many studies about their biological properties such as anti-inflammatory, anti-cancer, antioxidant, and anti-obesity effects [8]. *Commelina communis* L., Asian dayflower has been used in Chinese medicine [9,10], because it contains various chemical constituents such as flavonoids, terpenes, and, alkaloids, and has a variety of biological properties such as anti-inflammation, anti-obesity, and antioxidation. 1-deoxynojirimycin and (*2R*, *3R*, *4R*, *5R*)-2,5-*bis*(hydroxymethyl)-3,4-dihydroxypyrrolidine in *C. communis* inhibit α-glucosidase activity [9]. In a previous study, we found that *C. communis* tea decreased body weight gain in high-fat diet-fed mice, and that glucoluteolin (luteolin-7-*O*-glucoside) purified from *C. communis* suppressed glucose uptake in adipocytes [11]. 8-β-d-Glucopyranosyl-3′,4′,5,7-tetrahydroxyflavone (orientin; Figure 1A) is one of the major constituents in *C. communis*. Orientin has various medicinal properties [12] and has been reported to decrease intracellular triglyceride (TG) accumulation by inhibiting the expression of C/EBPα and PPARγ [13]. However, the molecular mechanism of inhibition of adipogenesis by orientin has never been elucidated.

In this study, we investigated the molecular mechanism by which orientin purified from *C. communis* suppresses adipogenesis in mouse adipocyte 3T3-L1 cells. We found, for the first time, that orientin reduces the intracellular lipid accumulation by suppressing PPARγ activity via downregulating the expression of C/EBPδ and inhibiting the phosphoinositide 3-kinase (PI3K)/Akt-forkhead transcription factors of class O1 (FOXO1) signaling in the early stage of adipocyte differentiation.

## 2. Materials and Methods 

### 2.1. Materials

3-Isobutyl-1-methylxanthine (IBMX), dexamethasone (Dex), insulin, and Oil Red O were purchased from Sigma (St. Louis, MO, USA). Akt Inhibitor X was obtained from Cayman Chemical (Ann Arbor, MI, USA). Anti-Akt, anti-phospho-Akt (p-Akt; Thr308), anti-phospho-FOXO1 (p-FOXO1; Ser256), anti-C/EBPα, and anti-hormone-sensitive lipase (HSL) polyclonal antibodies were from Cell Signaling (Danvers, MA, USA). Anti-PPARγ, anti-fatty acid synthase (FAS), and anti-FOXO1 polyclonal antibodies, and normal rabbit IgG, anti-mouse, anti-goat, or anti-rabbit IgG antibody conjugated with horseradish peroxidase (HRP) were from Santa Cruz Biotech. (Dallas, TX, USA). Anti-β-actin monoclonal and anti-glucose transporter 4 (GLUT4) polyclonal antibodies were from Sigma.

### 2.2. Purification of Orientin from C. communis

Orientin was purified from aerial parts of *C. communis* as described previously [11].

### 2.3. Cell Culture

Mouse adipocyte 3T3-L1 cells (Human Science Research Resources Bank, Osaka, Japan) were grown in Dulbecco’s Modified Eagle’s Medium (DMEM; Sigma) containing 10% (*v*/*v*) fetal bovine serum (CORNING, Corning, NY, USA) and antibiotics (Nacalai Tesque, Kyoto, Japan) at 37 °C in an atmosphere of 5% CO_2_. For adipocyte differentiation, at the day after reaching confluence (day 0), the medium was changed to DMEM containing insulin (10 μg/mL), 0.5 mM IBMX, and 1 μM Dex. At day 2, medium was changed to DMEM with insulin (10 μg/mL). The medium was changed every 2 days. The intracellular lipids were stained with Oil Red O as described previously [14].

### 2.4. Cytotoxicity Assay

Cell culture was performed for 6 days in medium with various concentrations of orientin (0–50 μM). The medium containing orientin was changed every 2 days. Cytotoxicity was measured by WST assay with a Cell Counting Kit-8 (DOJINDO, Kumamoto, Japan).

### 2.5. Measurement of Intracellular TG Level

Intracellular TG levels were measured by using a WAKO LabAssay Triglyceride Kit (Wako Pure Chemical, Osaka, Japan). Protein concentration was determined using a Pierce BCA Protein Assay Reagent (Thermo Fisher Scientific, Waltham, MA, USA) with bovine serum albumin to make the standard curve.

### 2.6. Quantification of mRNA Level by Quantitative PCR

Total RNA was prepared using ISOGEN (Nippon Gene, Tokyo, Japan). First-strand cDNA synthesis was carried out using total RNA (1 μg) and ReverTra Ace reverse transcriptase (Toyobo Osaka, Japan). The mRNA levels of the genes were assessed by quantitative PCR (qPCR: Applied Biosystems 7500 Real-Time PCR System; Thermo Fisher Scientific) using a *Power* SYBR Green PCR Master Mix (Thermo Fisher Scientific). The results were determined using the 2^−^^ΔΔCt^ method [15] and shown as the fold change relative to the control after normalizing to that of the TATA-binding protein (TBP) gene. The nucleotide sequences of the primers used were shown in Table 1.

### 2.7. Western Blot Analysis

Total cell lysates were prepared using ice-cold RIPA buffer (50 mM Tris-Cl, pH 8.0, 150 mM NaCl, 1% (*v*/*v*) NP-40, 0.5% (*w*/*v*) sodium deoxycholate, and 1% (*v*/*v*) Triton X-100) containing a protease inhibitor cocktail (Nacalai Tesque) and phosphatase inhibitors (1 mM Na_3_VO_4_, 50 μM Na_2_MoO_4_, and 1 mM NaF). Cell lysates were clarified by centrifugation (12,000× *g* for 20 min at 4 °C) and protein concentrations were measured as described above. Proteins were fractionated by SDS-polyacrylamide gel electrophoresis and transferred onto polyvinylpyrrolidone membranes (Immobilon P, Merck, Whitehouse Station, NJ, USA). The blots were treated for 1 h in blocking reagent; Blocking One or Blocking One-P (Nacalai Tesque). After washing the blots with Tris-buffered saline including 0.1% (*v*/*v*) Tween 20, they were incubated with a primary antibody. Then, the blots were incubated with a respective HRP-conjugated secondary antibody. Immunoreactive signals were visualized by using a Luminata Forte Western HRP Substrate (Merck) with a Lumino Image Analyzer LAS3000 (Fujifilm, Tokyo, Japan). Band intensity was measured by using Multi Gauge software (Fujifilm).

### 2.8. Lipolysis Assay

Lipolysis was evaluated by measuring the release of glycerol that hydrolyzed from TG, diglyceride, and monoglyceride [16]. 3T3-L1 cells were differentiated into adipocytes for 6 days in DMEM with or without orientin. At day 5, the medium was changed to phenol red-free DMEM (Sigma) including insulin with or without orientin. At day 6, the medium was collected and assayed for glycerol content by using a Free Glycerol Assay Reagent (Cayman Chemical).

### 2.9. Cell Proliferation Assay

Confluent 3T3-L1 cells were differentiated into adipocytes in medium containing orientin for the distinct time periods of the 6-days-adipogenesis. The cells were washed with PBS, and trypsinized. Cell number was measured with an Automated Cell Counter (TC-20, Bio-Rad, Hercules, CA, USA).

### 2.10. Chromatin Immunoprecipitation Assay

Chromatin immunoprecipitation (ChIP) assay was done as described previously [17]. Briefly, equal aliquots of chromatin supernatants were incubated with anti-FOXO1 antibody or rabbit normal IgG. After reverse crosslinking, the DNA was purified and used for PCR analysis. PCR was carried out using KOD FX DNA polymerase (Toyobo) and the primers; 5′-CCACTGGTGTGTATTTTACTGC-3′ and 5′-AAAATGGTGTGTCATAATGCTG-3′ in the following condition: after initial denaturation at 98 °C for 10 min, 32 cycles of 98 °C for 10 s, 55 °C for 15 s, and 72 °C for 30 s. The PCR products were analyzed using an agarose gel electrophoresis. The band intensity was measured and analyzed using ImageJ [18].

### 2.11. Statistical Analysis

Two groups were compared by using Student’s *t*-test. Two or more groups were compared using One-way analysis of variance and Tukey’s post-hoc test. Differences of *p* < 0.05 were considered to be statistically significant.

## 3. Results

### 3.1. Reduction of Intracellular TG Levels by Orientin

We examined the cytotoxicity of orientin on 3T3-L1 cells using WST assay. Cell culture was performed for 6 days in medium with various concentrations of orientin (0–50 μM). Orientin was nontoxic to 3T3-L1 cells at concentrations of <50 μM (Figure 1B). No morphological changes in the cells were observed in the microscopic analysis.

Next, we investigated the anti-adipogenic property of orientin on 3T3-L1 cells. The cells were differentiated into adipocytes for 6 days in medium with various concentrations of orientin (0, 10, 25, or 50 μM), and intracellular lipids were stained with Oil Red O. Oil Red O-stained lipid droplets in the cells were clearly increased in adipocyte differentiation, but they were reduced by orientin in a concentration-dependent manner (Figure 1C). Moreover, the elevated intracellular TG level during adipocyte differentiation was significantly lowered when treated with 50 μM orientin (Figure 1D). Based on these data, we used 50 μM orientin in subsequent studies.

### 3.2. Change in mRNA Level of Adipogenic, Lipogenic, and Lipolytic Genes by Orientin

**Figure 2:**
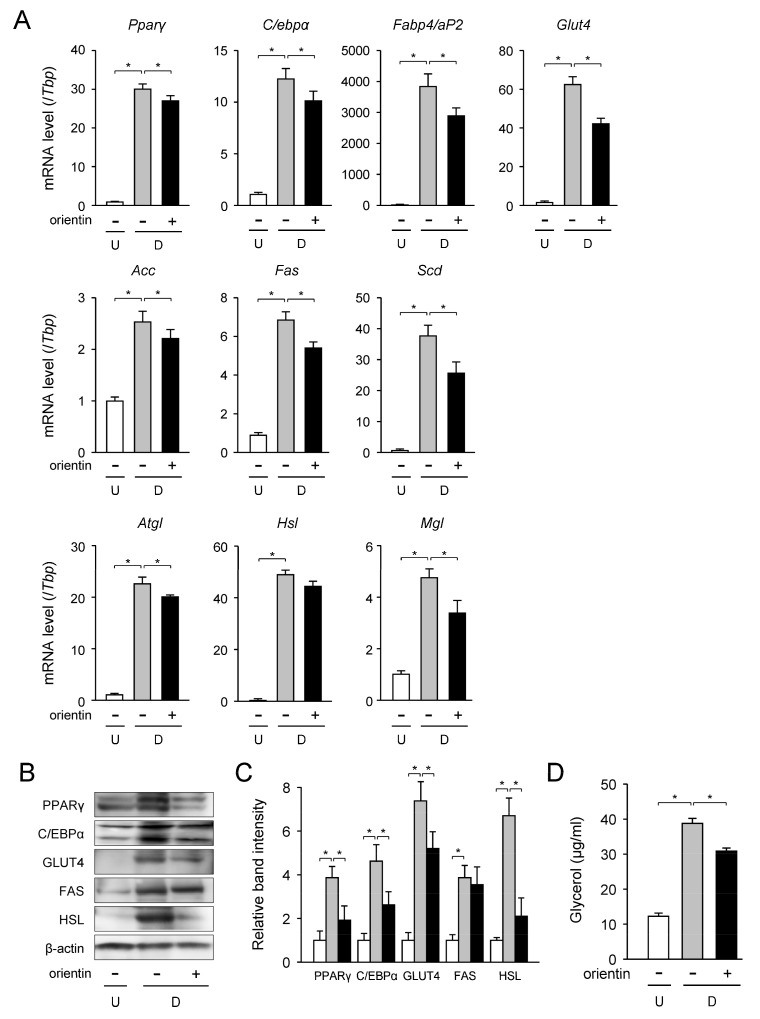
Change in expression level of adipogenic, lipogenic, and lipolytic genes/proteins by orientin. (**A**) The mRNA levels of the adipogenic, lipogenic, and lipolytic genes in orientin-treated 3T3-L1 cells. Undifferentiated 3T3-L1 cells (U; white columns) were differentiated into adipocytes (D; gray columns) for 6 days in medium with orientin (50 μM; black columns). The data are represented as the means ± S.D. from three experiments. * *p* < 0.01, as shown by the brackets; (**B**) Change in protein expression level by orientin. 3T3-L1 cells were caused to differentiate as described in the legend of Figure 2A. For Western blot analysis, 15 μg protein was loaded in each lane. Data are the representative of three experiments. Each band intensity was normalized with that of β-actin; (**C**) Measurement of intensity of band shown in Figure 2B. Data are shown as the means ± S.D. from three experiments. * *p* < 0.01, as shown by the brackets; (**D**) Decrease in glycerol release by orientin. Data are represented as the means ± S.D. from three experiments. * *p* < 0.01, as shown by the brackets.

Transcription levels of the adipogenic, lipogenic, and lipolytic genes in the orientin-treated differentiated 3T3-L1 cells were measured by qPCR. The mRNA levels of the adipogenic *Pparγ*, *C/ebpα*, fatty acid binding protein 4 (*Fabp4, aP2*), and *Glut4* genes were enhanced about 30-, 12-, 3800-, and 62-fold, respectively, during adipogenesis (Figure 2A). While, when 3T3-L1 cells were caused to differentiate in medium with orientin, these mRNA levels were lowered about 10%, 21%, 25%, and 32%, respectively, of those of the differentiated cells (Figure 2A).

Moreover, the mRNA levels of the acetyl-CoA carboxylase (*Acc*), *Fas*, and stearoyl-CoA desaturase (*Scd*) genes in lipogenesis were measured by qPCR. 3T3-L1 cells were differentiated into adipocytes for 6 days in medium containing orientin (0–50 μM). The expression levels of the *Acc*, *Fas*, and *Scd* genes were elevated about 2.6-, 6.9-, and 38-fold, respectively, as compared with the undifferentiated cells (Figure 2A). In contrast, these mRNA levels were decreased approximately 17%, 19%, and 34%, respectively, when treated with orientin (Figure 2A).

Intracellular TG is hydrolyzed into fatty acids and glycerol through the activities of three lipases; adipocyte TG lipase (ATGL), HSL, and monoacyl glyceride lipase (MGL) [19]. Then, we investigated the change in the mRNA level of these three lipase genes when treated with orientin. The transcription levels of the *Atgl*, *Hsl*, and *Mgl* genes in the differentiated cells were enhanced about 22-, 48-, and 4.7-fold, respectively, as compared with the undifferentiated cells (Figure 2A); however orientin reduced the expression levels of these genes by approximately 14%, 11%, and 23%, respectively (Figure 2A).

The protein levels of some of these genes were examined by Western blot analysis. The protein levels of adipogenic PPARγ, C/EBPα, GLUT4, lipogenic FAS, and lipolytic HSL were elevated during adipogenesis (Figure 2B,C); however, these enhancements were significantly lowered by the treatment with orientin (Figure 2B,C). Meanwhile, the expression level of FAS tended to decrease by the treatment with orientin.

Furthermore, we measured the levels of glycerol released from adipocytes. The glycerol release levels were elevated during adipogenesis (Figure 2D). This adipogenesis-mediated increase in the level of glycerol release was reduced about 21% of the differentiated cells by treating with orientin (Figure 2D). These results reveal that orientin suppressed the expression of adipogenic, lipogenic, and lipolytic genes/proteins in 3T3-L1 cells.

### 3.3. Change in Expression Level of TG Synthetic Enzyme Genes by Orientin

TG is synthesized through four steps from fatty acyl-CoA and glycerol-3-phosphate [20]. The initial step is to form lysophosphatidic acid (1-acylglycerol-3-phosphate) from glycerol-3-phosphate catalyzed by glycerol-3-phosphate acyltransferase (GPAT). Lysophosphatidic acid is converted to phosphatidic acid (1,2-diacylglycerol-3-phosphate) by catalyzing with 1-acylglycerol-3-phosphate acyltransferase (AGPAT). The phosphatidic acid is then converted to 1,2-diacylglycerol (1,2-DAG) by phosphatidate phosphatase-1 (PAP: lipin-1). Finally, 1,2-DAG is acylated to produce TG by diacylglycerol acyltransferase (DGAT). These enzymes involved in the TG synthetic pathway have also some isozymes [20].

**Figure 3:**
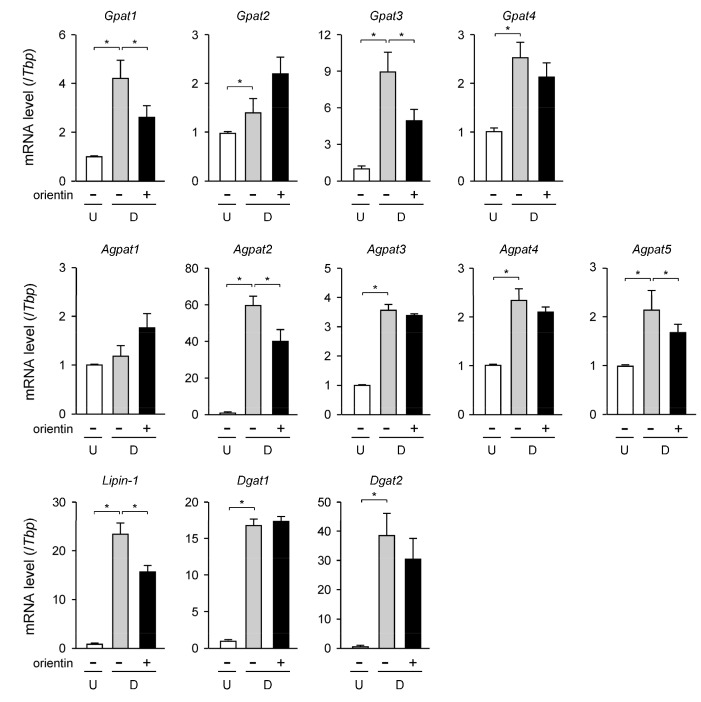
Effect on expression level of TG synthetic enzyme genes by orientin. 3T3-L1 cells were caused to differentiate into adipocytes as described in the legend of Figure 2A. Data are shown as the means ± S.D. from three experiments. * *p* < 0.01, as shown by the brackets.

We carried out qPCR analysis to investigate the change in the transcription level of the TG synthetic enzyme genes in the orientin-treated 3T3-L1 cells. The expression levels of the *Gpat1*, *Gpat2*, *Gpat3*, and *Gpat4* genes were all enhanced approximately 4.3-, 1.4-, 8.8- and 2.5-fold, respectively, in adipogenesis (Figure 3). Among them, the mRNA levels of the *Gpat1* and *Gpat3* genes were reduced about 29% and 41%, respectively, by the treatment with orientin (Figure 3); however, the expression levels of the *Gpat2* and *Gpat4* genes were not changed when treated with orientin (Figure 3). The transcription levels of the *Agpat2*, *Agpat3*, *Agpat4*, and *Agpat5* genes were enhanced about 59-, 3.5-, 2.3-, and 2.1-fold, respectively, in adipogenesis (Figure 3). The expression levels of the *Agpat2* and *Agpat5* genes were decreased to approximately 66% and 81%, respectively, when the cells were differentiated in medium containing orientin (Figure 3). In contrast, orientin did not affect to the mRNA levels of the *Agpat3* and *Agpat4* genes (Figure 3), and the transcription levels of the *Agpat1* gene were not altered in adipogenesis, even in the presence of orientin (Figure 3). The expression levels of the *Lipin-1* gene were elevated about 23-fold during adipogenesis, and its expression was lowered about 30% by the treatment with orientin (Figure 3). In contrast, the mRNA levels of the *Dgat1* and *Dgat2* genes were not affected by treating with orientin although the expression level of these genes was enhanced approximately 17- and 38-fold, respectively, in adipogenesis (Figure 3). These results mean that the expression levels of some TG biosynthetic enzyme genes were suppressed by orientin in adipocytes.

### 3.4. Decrease in Expression Level of C/ebpδ Gene by Orientin in Early Stage of Adipogenesis

**Figure 4:**
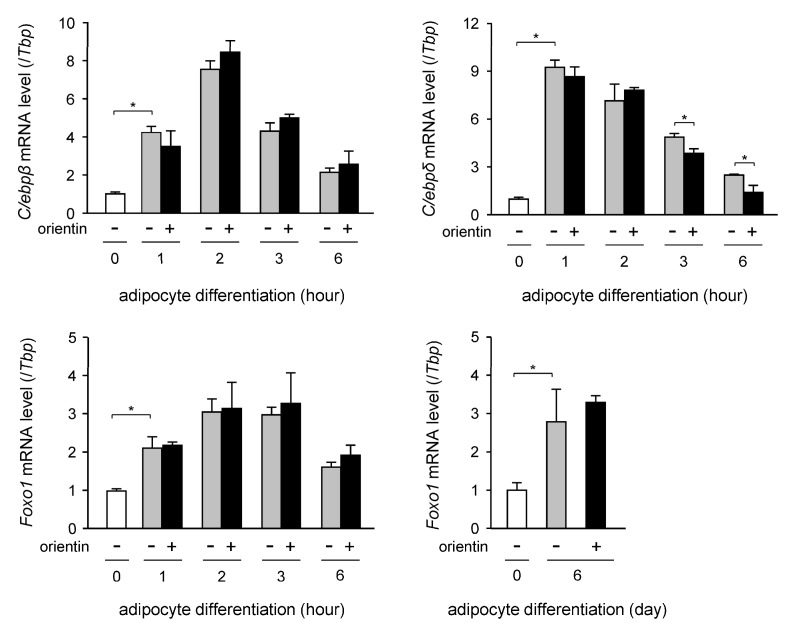
Expression profile of the *C/ebpβ*, *C/ebpδ*, and *Foxo1* genes in early stage of adipogenesis 3T3-L1 cells (white columns) were allowed to differentiate into adipocyte (differentiated cells: gray columns) for the indicated time (h:h) in medium containing orientin (50 μM; black columns). The expression level of the *C/ebpβ*, *C/ebpδ*, and *Foxo1* genes was measured by qPCR. Data are the means ± S.D. from three experiments. * *p* < 0.01, as shown by the brackets.

To elucidate the molecular mechanism of the orientin-mediated repression of adipocyte differentiation, we investigated the change in the expression profile of the *C/ebpβ*, *C/ebpδ*, and *Foxo1* genes in the early stage of adipogenesis (0–6 h after the initiation of adipogenesis). The expression level of the *C/ebpβ* gene was transiently enhanced, peaking at 2 h after the initiation of adipogenesis and gradually decreased after that (Figure 4). The expression level of the *C/ebpδ* gene was also induced at 1 h after the start of adipogenesis and gradually decreased during adipogenesis (Figure 4). Moreover, the expression level of the *C/ebpδ* gene was decreased at 3 and 6 h after starting of adipogenesis by the treatment with orientin (Figure 4). The transcription level of the *Foxo1* gene was enhanced in the early stage of adipogenesis (Figure 4), but its expression level was not affected by the treatment with orientin in either the early stage of adipogenesis or the 6-days-adipogeneis (Figure 4).

### 3.5. Repression of Dex-Mediated Activation of C/ebpδ Expression by Orientin

**Figure 5:**
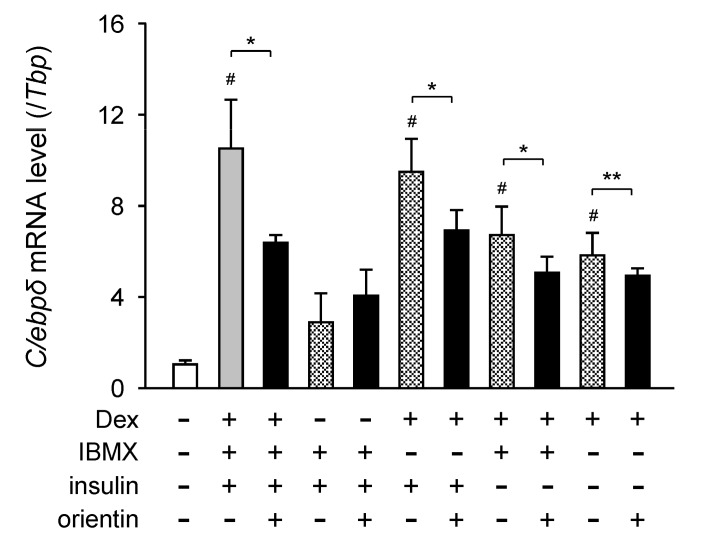
Reduction of dexamethasone (Dex)-mediated enhancement of *C/ebpδ* expression by orientin. 3T3-L1 cells were differentiated into adipocytes for 6 h in medium with (+) or without (−) 1 μM Dex, 0.5 mM IBMX, 10 μg/mL insulin, and/or 50 μM orientin. Data are shown as the means ± S.D. from three experiments. * *p* < 0.01 and ** *p* < 0.05, as shown by the brackets. ^#^ *p* < 0.01, as compared with the vehicle-treated cells (white column).

Dex is known to be involved in the activation of the expression of the C/*C/ebp* genes in adipocytes [21]. To examine the regulation of Dex-mediated activation of *C/ebpδ* expression, 3T3-L1 cells were differentiated into adipocytes for 6 h in medium with Dex, IBMX, insulin, and/or orientin. The expression level of the *C/ebpδ* gene was not enhanced when the cells were cultured in medium with IBMX and insulin without Dex (Figure 5). Whereas, its expression level was elevated in medium containing Dex (Figure 5). Moreover, the Dex-mediated elevation of the expression of the *C/ebpδ* gene was reduced by co-treating with orientin (Figure 5). However, the *C/ebpδ* mRNA levels in the cells cultured in medium containing IBMX and/or insulin without Dex were not affected by orientin (Figure 5). These results indicate that orientin repressed the Dex-induced expression of the *C/ebpδ* gene in the early stage of adipogenesis of 3T3-L1 cells.

### 3.6. Repression of Activation of PI3K/Akt-FOXO1 Signaling by Orientin

**Figure 6:**
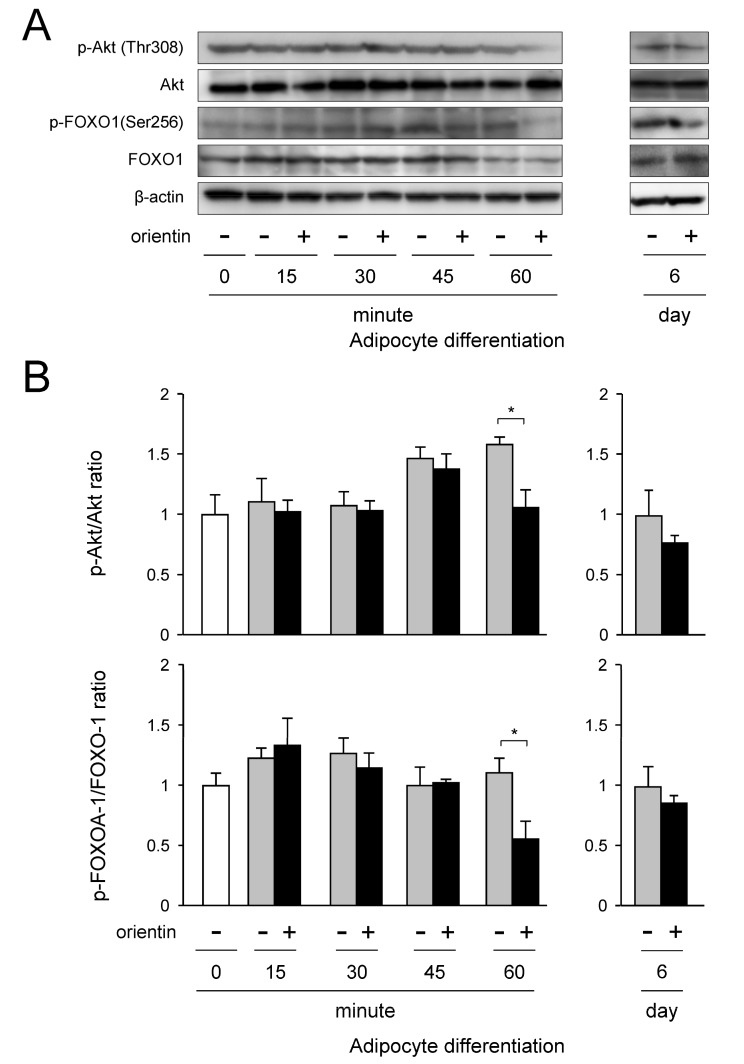
Reduced phosphorylation of Akt and FOXO1 by orientin in early stage of adipogenesis. (**A**) Western blot analysis. 3T3-L1 cells were differentiated into adipocytes for the indicated time periods in medium with orientin (0 or 50 μM). For Western blot analysis, 15 μg protein was loaded in each lane. Data are the representative of three experiments; (**B**) Ratio of p-Akt/Akt and p-FOXO1/FOXO1 levels. The expression levels shown in Figure 6A were measured. Before calculation of ratio, each band intensity was normalized with that of β-actin. Data are shown as the means ± S.D. from three experiments. * *p* < 0.01, as shown by the brackets.

The PI3K/Akt signaling is activated by insulin [22]. We examined how insulin signaling is regulated adipogenesis in 3T3-L1 cells. Expression of Akt was detected in the undifferentiated cells (0 min), and the expression level of Akt was maintained fairly consistently in adipocyte differentiation (Figure 6A,B). Although Akt was continuously phosphorylated in adipogenesis, its phosphorylation level was diminished at 60 min after initiating the treatment with orientin (Figure 6A,B). Phosphorylation of FOXO1 is activated by phosphorylated Akt [23,24]. FOXO1 was continuously expressed and phosphorylated in adipogenesis (Figure 6A,B). However, when the 3T3-L1 cells were differentiated in medium with orientin, the phosphorylation level of FOXO1 decreased at 60 min after starting the treatment (Figure 6A,B). In addition, phosphorylation level of Akt and FOXO1 was not altered even when the cells were differentiated for 6 days in medium containing orientin (Figure 6A,B). These results reveal that orientin decreased the phosphorylation of Akt and FOXO1 in the early stage of adipogenesis of 3T3-L1 cells.

### 3.7. Stage-Specific Repression of Adipogenesis by Orientin

**Figure 7:**
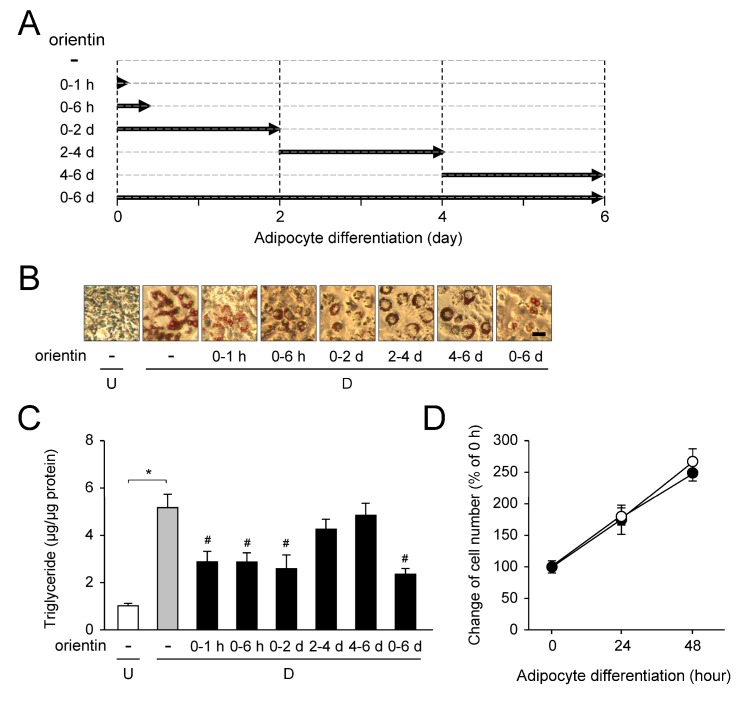
Stage-specific effect of orientin in adipocyte differentiation. (**A**) Addition of orientin at the distinct time periods; 0–1 h (0–1 h), 0–6 h (0–6 h), 0–2 days (0–2 d), 2–4 days (2–4 d), 4–6 days (4–6 d), or 0–6 days (0–6 d) during the 6-days-adipogenesis; (**B**) Oil Red O-stained intracellular lipids in medium with stage-specific addition of orientin. 3T3-L1 cells (undifferentiated cells: U; white column) were allowed to differentiate into adipocytes (differentiated cells: D) for 6 days in the absence (gray column) or presence (50 μM; black columns) of orientin. Bar = 50 μm; (**C**) Intracellular TG level. 3T3-L1 cells were differentiated as described in the legend of Figure 7B. Data are the means ± S.D. from three experiments. * *p* < 0.01, as shown by the brackets. ^#^ *p* < 0.01, as compared with the vehicle-treated differentiated cells (gray column); (**D**) Effect of orientin to mitotic clonal expansion. Confluent 3T3-LI preadipocytes were differentiated in medium without (closed circles) or with orientin (50 μM; open circles) for 48 h. Cell number was counted by Cell Counter. The data are shown as the means ± S.D. from three experiments.

We investigated the suppression effect of orientin in the early stages of adipogenesis, because orientin repressed the expression of C/EBPδ that is an important transcription factor in the early stage of adipogenesis (Figure 4) and decreased the phosphorylation of Akt and FOXO1 at 60 min (1 h) after the initiation of adipogenesis (Figure 6A,B). 3T3-L1 cells were differentiated into adipocytes for 6 days in medium containing orientin at the indicated period (0–1 h, 0–6 h, 0–2 d, 2–4 d, 4–6 d, or 0–6 d; Figure 7A). When orientin was added during 0–1 h, 0–6 h, or 0–2 d of 6-days-adipogenesis, Oil Red O-stained intracellular lipid accumulation and intracellular TG level were decreased as compared with the vehicle-treated differentiated cells (Figure 7B,C). Moreover, these levels were more reduced when the cells were differentiated in medium containing orientin for all 6 days of adipogenesis (Figure 7A,B). However, when orientin was added during 2–4 d or 4–6 d of 6-days-adipogenesis, the intracellular lipid accumulation was a little decreased as compared with that of the vehicle-treated differentiated cells (Figure 7B,C).

In addition, when 3T3-L1 preadipocytes were differentiated into adipocytes for 48 h, cell number was increased more than 2.5-fold (Figure 7D). However, even when the cells were differentiated in medium containing orientin, cell number was almost the same as that of the vehicle treated differentiated cells (Figure 7D). These results suggest that orientin did not affect to the cell proliferation in the mitotic clonal expansion in the early stage of adipogenesis.

### 3.8. Decrease of Binding of FOXO1 to Pparγ Promoter by Orientin

**Figure 8:**
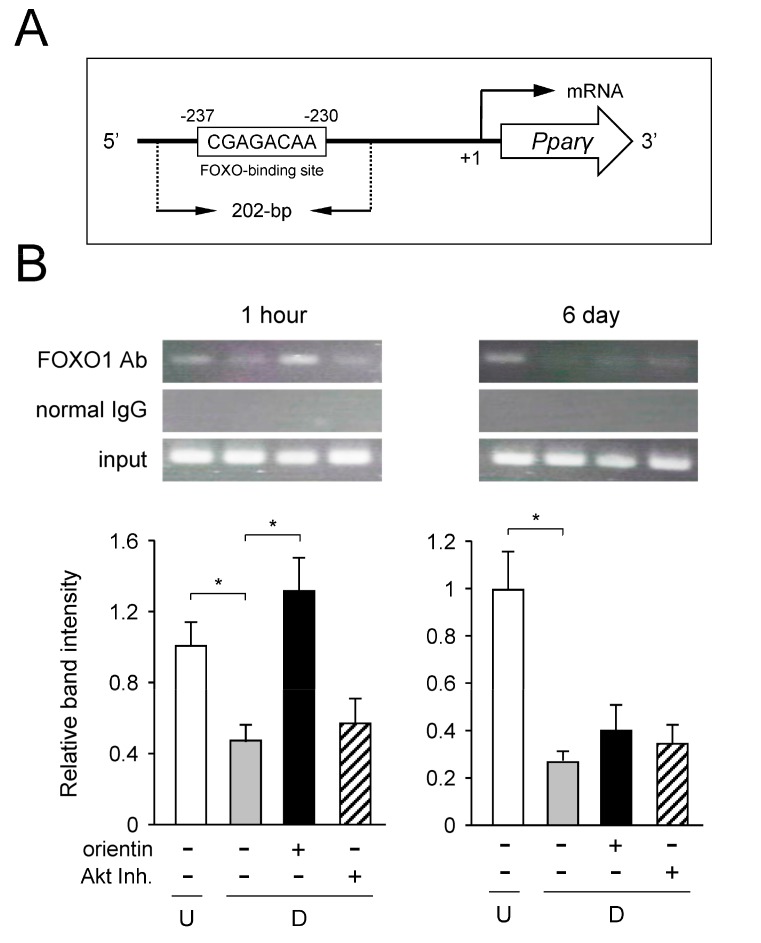
Increased binding of FOXO1 to *Pparγ* promoter by orientin. (**A**) Schematic representation of the FOXO-binding site in the mouse *Pparγ* promoter. The amplicon obtained from the ChIP assay was also shown; (**B**) ChIP assay of the FOXO-binding site in mouse *Pparγ* promoter in 3T3-L1 cells. Undifferentiated cells (U; white columns) were caused to differentiate into adipocytes (D; gray columns) for 1 h or 6 days in medium with orientin (50 μM; black columns), or Akt inhibitor X (10 μM, Akt Inh.; hatched columns). The input control (input) indicates a small aliquot from prior to immunoprecipitation. Data represent as the mean ± S.D. * *p <* 0.01, as shown by the brackets. Ab: antibody.

The FOXO-binding site is located at −237 in the proximal promoter region of the mouse *Pparγ* gene [14]. In a ChIP assay, the expected size of the PCR fragments containing the FOXO-binding site is 202-bp (Figure 8A). Amplified PCR fragments were detected in the undifferentiated cells at both 1 h and 6 days (Figure 8B). Its signal intensity was diminished by differentiation into adipocytes at 1 h (Figure 8B), indicating that binding of FOXO1 to the *Pparγ* promoter was decreased. In contrast, the intensity of PCR fragments was enhanced by the treatment with orientin or Akt inhibitor (Akti) at both 1 h and 6 days (Figure 8B). No amplified signals were detected when rabbit normal IgG was utilized instead of anti-FOXO1 antibody (Figure 8B). PCR fragments of the expected size were detected in all of the input samples (Figure 8B). These results indicate that the adipocyte differentiation-dependent decrease of binding of FOXO1 to the *Pparγ* promoter was negated by orientin through suppressing the activation of the PI3K/Akt-FOXO1 signaling in 3T3-L1 cells.

## 4. Discussion

Adipogenesis is a unique process for accumulation of lipids as TG in adipocytes to maintain energy homeostasis in the body [4]. However, the excess accumulation of lipids in adipocytes results in obesity, which can cause various metabolic diseases such as cardiovascular diseases and type II diabetes [1,2,3]. Thus, the protection and elimination of obesity are important issues for human health. Although anti-obesity drugs have been developed, their use is limited to patients with a body mass index >30 kg/m^2^ [25,26]. Moreover, some of these anti-obesity drugs have emotional or physical side effects such as cardiovascular issues [25,26,27].

**Figure 9:**
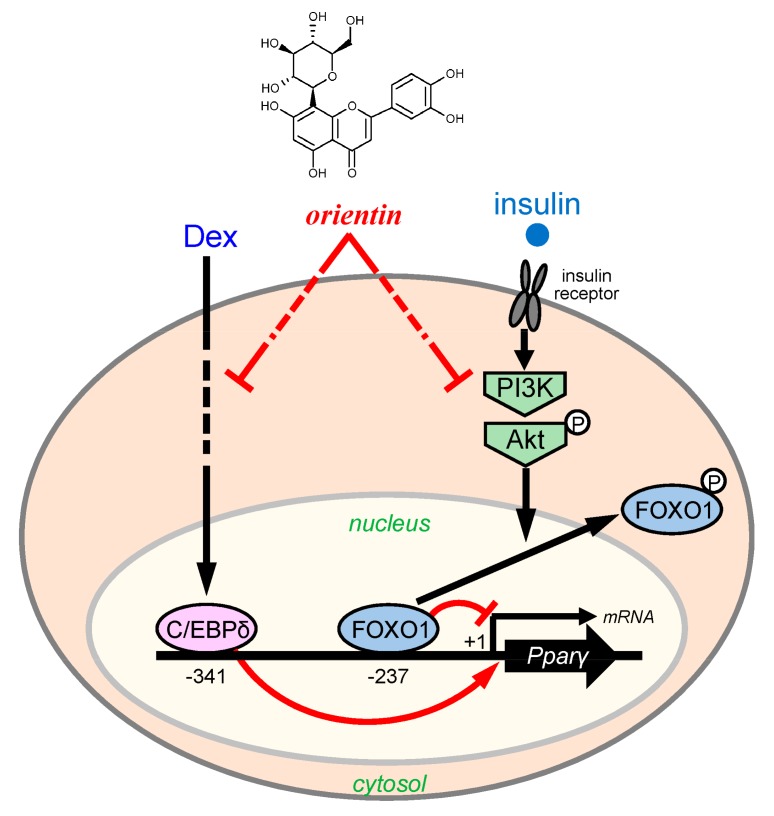
Proposed mechanism of orientin-mediated suppression of adipogenesis in 3T3-L1 cells. C/EBP- and FOXO-binding elements were shown at −341 and −237, respectively. Transcription initiation site of the *Pparγ2* mRNA was defined as +1.

Some phenolic flavonoids in vegetables and fruits have a number of physiological effects on various diseases [8,28]. Most of these natural products show weaker beneficial effects than pharmaceutical medicines on diseases, but they have less inconvenient and fewer side effects. Some natural products such as (−)-epigallocatechin gallate, genistein, avicularin, baicalein, and apigenin inhibit adipogenesis [8]. Recently, we identified that the tea of *C. communis* decreased body weight gain in mice, and that glucoluteolin, one of major constituents of *C. communis* lowered the intracellular lipid accumulation by repressing the incorporation of glucose into adipocytes [11]. In this study, we provided evidence that orientin from *C. communis* lowered the intracellular lipid accumulation by decreasing fatty acid and TG synthesis through reducing C/EBPδ expression and inhibition of the PI3K/Akt-FOXO1 signaling in the early stage of adipogenesis (Figure 9). However, we have not yet investigated the transportation of orientin into the cells and the modification and metabolism of orientin in the medium and in the cells. Thus, we have to investigate the modification and metabolism of orientin for further in vivo study.

In mammals, long-chain fatty acids are synthesized from acetyl-CoA by acting with ACC and FAS, and are desaturated by SCD. The expression of the *Scd* gene is activated by PPARγ in adipocytes [29]. Moreover, synthesis of TG is important for nutrient utilization and energy storage. Intracellular TG is synthesized through four enzymatic steps that include several isoforms of GPAT, AGPAT, lipin-1, and DGAT by connecting fatty acyl-CoA with glycerol backbone [30]. Ablation of some of these TG synthetic enzymes decrease body fat by reducing the accumulation of intracellular lipids in adipocytes [20,31]. Furthermore, the expression of the GPAT3 is activated by PPARγ [32]. Therefore, fatty acid and TG synthesis are regulated by PPARγ in adipocytes. In this study, we found that orientin decreased the expression of several genes that are involved in fatty acid and TG synthesis (Figure 2A,B and Figure 3) as well as PPARγ in adipocytes (Figure 2A,B). Thus, orientin suppresses fatty acid and TG synthesis through reduction of PPARγ activity in adipocytes.

The regulatory mechanism of adipogenesis is so complex and a number of transcription factors are involved in this regulation. PPARγ, a critical transcription factor, plays an important role in the regulation of adipogenesis by controlling the expression of a number of adipogenesis-related genes. While, the expression of the *Pparγ* gene is regulated by several transcription factors in adipocytes. Moreover, C/EBPβ and C/EBPδ, transcription factors that are activated in the early stage of adipogenesis, enhance the expression of the *Pparγ* gene [33,34,35]. Orientin decreased the expression of the *C/ebpδ* gene in the early stage of adipogenesis (Figure 4). Expression of the *C/ebpδ* gene in the early stage of adipogenesis is activated by Dex, which is included in the adipocyte-differentiation cocktail. Only Dex, but not IBMX and insulin enhanced the expression of the *C/ebpδ* gene in the early stage of adipogenesis (Figure 5). Furthermore, Dex-mediated induction of the expression of the *C/ebpδ* gene was reduced by co-treating with orientin (Figure 5). It is reported that Dex is essential for the activation of the early stage of adipogenesis by enhancing the expression of C/EBPβ and C/EBPδ [21]. However, orientin decreased the transcription of C/EBP*C/ebpδ,* but not *C/ebpβ* in the early stage of adipogenesis (Figure 4). Although Dex acts by binding with glucocorticoid receptor (GR), orientin did not antagonize GR function. The regulatory mechanism of suppression of the Dex-activated expression of the *C/EBPδ* gene by orientin should be further elucidated. Moreover, it was reported that a flavonoid scutellarin inhibits adipogenesis by upregulating PPARα expression through acting as a PPARα agonist [36]. Although the expression level of the *Pparα* gene was not affected by orientin, the modulation of PPARα activity should be investigated.

Insulin signaling is important in the regulation of adipocyte differentiation [37]. In this signaling, the regulation of PI3K/Akt is critical in adipocyte differentiation [38,39]. FOXO proteins have a winged helix DNA binding domain and are involved in the regulation of metabolism, cell fate decision, apoptosis, and cell differentiation [40] through insulin-mediated PI3K/Akt signaling. Activated (phosphorylated) Akt phosphorylates FOXO1 at three Ser/Thr residues [41], leading to the translocation of FOXO1 from nucleus to cytoplasm, resulting in clearance of FOXO1-mediated inhibition of transcription of the *Pparγ* gene [40,42]. In this study, orientin repressed the phosphorylation of Akt in the early stage of adipogenesis, followed by decreased phosphorylation of FOXO1 (Figure 6A,B), leading to the reduction of binding of FOXO1 to the *Pparγ* promoter by repressing the PI3K/Akt signaling (Figure 8A,B).

## 5. Conclusions

We showed that a flavonoid orientin from *C. communis* decreased the lipid accumulation in mouse adipocytes. Orientin decreased the expression of C/EBPδ and suppressed the PI3K/Akt-FOXO1 signaling in the early stage of adipogenesis, leading to reduced expression of the *Pparγ* gene. Thus, orientin has the potential to suppress an increase of cell size of adipocytes. We should further elucidate the mechanism by which orientin suppresses Dex-activated PPARγ expression and conduct an in vivo study to evaluate the anti-obesity properties of orientin.

## Figures and Tables

**Table 1:** Nucleotide Sequences of Primers Used in qPCR.

Gene	Accession No. *	Forward Primer	Reverse Primer
*Pparγ*	NM_011146	5′-CAAGAATACCAAAGTGCGATCAA-3′	5′-GAGCTGGGTCTTTTCAGAATAATAAG-3′
*C/Eebpα*	NM_007678	5′-CTGGAAAGAAGGCCACCTC-3′	5′-AAGAGAAGGAAGCGGTCCA-3′
*C/ebpβ*	NM_009883	5′-TGATGCAATCCGGATCAA-3′	5′-CACGTGTGTTGCGTCAGTC-3′
*C/ebpδ*	NM_007679	5′-GGGCAGTGGAGTAAGGTACAGA-3′	5′-GCACTGTCACCCATACAATGTT-3′
*Fabp4(aP2)*	NM_024406	5′-CAGCCTTTCTCACCTGGAAG-3′	5′-TTGTGGCAAAGCCCACTC-3′
*Glut4*	NM_009204	5′-GACGGACACTCCATCTGTTG-3′	5′-GCCACGATGGAGACATAGC-3′
*Acc*	NM_133360	5′-GCGTCGGGTAGATCCAGTT-3′	5′-CTCAGTGGGGCTTAGCTCTG-3′
*Fas*	NM_007988	5′-GTTGGGGGTGTCTTCAACC-3′	5′-GAAGAGCTCTGGGGTCTGG-3′
*Scd*	NM_009127	5′-CGTCTGGAGGAACATCATTCT-3′	5′-CAGAGCGCTGGTCATGTAGT-3′
*Atgl*	NM_001163689	5′-TGACCATCTGCCTTCCAGA-3′	5′-TGTAGGTGGCGCAAGACA-3′
*Hsl*	NM_010719	5′-GCACTGTGACCTGCTTGGT-3′	5′-CTGGCACCCTCACTCCATA-3′
*Mgl*	NM_011844	5′-TCGGAACAAGTCGGAGGT-3′	5′-TCAGCAGCTGTATGCCAAAG-3′
*Gpat1*	NM_008149	5′-GGAAGGTGCTGCTATTCCTG-3′	5′-TGGGATACTGGGGTTGAAAA-3′
*Gpat2*	NM_001081089	5′-GCTGCCAGACCTGTACTCCT-3′	5′-AGCCCAGGTCCATTATGCTT-3′
*Gpat3*	NM_172715	5′-GTGCTGGGTGTCCTAGTGC-3′	5′-AAGCTGATCCCAATGAAAGC-3′
*Gpat4*	NM_018743	5′-GAGTGCTGATTCGGTATTGCT-3′	5′-CACTACCAAGAGGCCAATCC-3′
*Agpat1*	NM_018862	5′-CTGTCTGTGGAAGCACCTTG-3′	5′-GCAGAACCACAGGGTGGA-3′
*Agpat2*	NM_026212	5′-AAGACGAAGCTCTTCACCTCA-3′	5′-TCTGTCAGACCATTGGTAGGG-3′
*Agpat3*	NM_053014	5′-CTGCCCCCACTCAAGTACC-3′	5′-TCAGGGTCACGTCATAGATAGC-3′
*Agpat4*	NM_026644	5′-ACGCTGACTGCTACGTTCG-3′	5′-TGTGTAACCAGGCAGAGCAC-3′
*Agpat5*	NM_026792	5′-CTAGCGAATCATCAAAGCACA-3′	5′-TCTTTCAGTACGTAGCGCACA-3′
*lipin-1*	NM_015763	5′-TCCCAGTTCGGACAGAGAAT-3′	5′-GGGAGTCCTCTGGCAATCTA-3′
*Dgat1*	NM_010046	5′-GCCCCATGCGTGATTATT-3′	5′-TCTGTCAGGGCACCCACT-3′
*Dgat2*	NM_026384	5′-GGCGCTACTTCCGAGACTAC-3′	5′-TGGTCAGCAGGTTGTGTGTC-3′
*Foxo1*	NM_019739	5′-CTTCAAGGATAAGGGCGACA-3′	5′-GACAGATTGTGGCGAATTGA-3′
*Tbp*	NM_013684	5′-GTGATGTGAAGTTCCCCATAAGG-3′	5′-CTACTGAACTGCTGGTGGGTCA-3′

* DDBJ/ENA/GenBank database.

## References

[1] Finucane M.M., Stevens G.A., Cowan M.J., Danaei G., Lin J.K., Paciorek C.J., Singh G.M., Gutierrez H.R., Lu Y., Bahalim A.N. (2011). National, regional, and global trends in body-mass index since 1980: Systematic analysis of health examination surveys and epidemiological studies with 960 country-years and 9.1 million participants. Lancet.

[2] Attie A.D., Scherer P.E. (2009). Adipocyte metabolism and obesity. J. Lipid Res..

[3] Cornier M.A., Dabelea D., Hernandez T.L., Lindstrom R.C., Steig A.J., Stob N.R., van Pelt R.E., Wang H., Eckel R.H. (2008). The metabolic syndrome. Endocr. Rev..

[4] Kershaw E.E., Flier J.S. (2004). Adipose tissue as an endocrine organ. J. Clin. Endocrinol. Metab..

[5] Matsuzawa Y. (2006). The metabolic syndrome and adipocytokines. FEBS Lett..

[6] Lefterova M.I., Lazar M.A. (2009). New developments in adipogenesis. Trends Endocrinol. Metab..

[7] Rosen E.D., Hsu C.H., Wang X., Sakai S., Freeman M.W., Gonzalez F.J., Spiegelman B.M. (2002). C/EBPα induces adipogenesis through PPARγ: A unified pathway. Genes Dev..

[8] Di Carlo G., Mascolo N., Izzo A.A., Capasso F. (1999). Flavonoids: Old and new aspects of a class of natural therapeutic drugs. Life Sci..

[9] Shibano M., Kakutani K., Taniguchi M., Yasuda M., Baba K. (2008). Antioxidant constituents in the dayflower (*Commelina communis* L.) and their α-glucosidase-inhibitory activity. J. Nat. Med..

[10] Zhang G.B., Tian L.Q., Li Y.M., Liao Y.F., Li J., Bing F.H. (2013). Protective effect of homonojirimycin from *Commelina communis* (dayflower) on influenza virus infection in mice. Phytomedicine.

[11] Nagai S., Wakai E., Shibano M., Fujimori K. (2016). Anti-obesity effects of Asian dayflower, *Commelina communis*, in mice with high-fat diet-induced obesity and in 3T3-L1 cells. J. Funct. Foods.

[12] Lam K.Y., Ling A.P., Koh R.Y., Wong Y.P., Say Y.H. (2016). A review on medicinal properties of orientin. Adv. Pharmacol. Sci..

[13] Kim J., Lee I., Seo J., Jung M., Kim Y., Yim N., Bae K. (2010). Vitexin, orientin and other flavonoids from Spirodela polyrhiza inhibit adipogenesis in 3T3-L1 cells. Phytother. Res..

[14] Baba S., Ueno Y., Kikuchi T., Tanaka R., Fujimori K. (2016). A limonoid Kihadanin B from immature *Citrus unshiu* peels suppresses adipogenesis through repression of the Akt-FOXO1-PPARγ axis in adipocytes. J. Agric. Food Chem..

[15] Livak K.J., Schmittgen T.D. (2001). Analysis of relative gene expression data using real-time quantitative PCR and the 2^−ΔΔ*C*^_T_ method. Methods.

[16] Ahmadian M., Wang Y., Sul H.S. (2010). Lipolysis in adipocytes. Int. J. Biochem. Cell. Biol..

[17] Kobayashi T., Fujimori K. (2012). Very long-chain-fatty acids enhance adipogenesis through coregulation of Elovl3 and PPARγ in 3T3-L1 cells. Am. J. Physiol. Endocrinol. Metab..

[18] Schneider C.A., Rasband W.S., Eliceiri K.W. (2012). NIH Image to ImageJ: 25 years of image analysis. Nat. Methods.

[19] Duncan R.E., Ahmadian M., Jaworski K., Sarkadi-Nagy E., Sul H.S. (2007). Regulation of lipolysis in adipocytes. Annu. Rev. Nutr..

[20] Takeuchi K., Reue K. (2009). Biochemistry, physiology, and genetics of GPAT, AGPAT, and lipin enzymes in triglyceride synthesis. Am. J. Physiol. Endocrinol. Metab..

[21] Cao Z., Umek R.M., McKnight S.L. (1991). Regulated expression of three C/EBP isoforms during adipose conversion of 3T3-L1 cells. Genes Dev..

[22] Mackenzie R.W., Elliott B.T. (2014). Akt/PKB activation and insulin signaling: A novel insulin signaling pathway in the treatment of type 2 diabetes. Diabetes Metab. Syndr. Obes..

[23] Wang Y., Zhou Y., Graves D.T. (2014). FOXO transcription factors: Their clinical significance and regulation. Biomed. Res. Int..

[24] Engelman J.A. (2009). Targeting PI3K signalling in cancer: Opportunities, challenges and limitations. Nat. Rev. Cancer.

[25] Cunningham J.W., Wiviott S.D. (2014). Modern obesity pharmacotherapy: Weighing cardiovascular risk and benefit. Clin. Cardiol..

[26] Jensen M.D., Ryan D.H., Apovian C.M., Ard J.D., Comuzzie A.G., Donato K.A., Hu F.B., Hubbard V.S., Jakicic J.M., Kushner R.F. (2014). 2013 AHA/ACC/TOS guideline for the management of overweight and obesity in adults: A report of the American College of Cardiology/American Heart Association Task Force on Practice Guidelines and The Obesity Society. Circulation.

[27] Dietrich M.O., Horvath T.L. (2012). Limitations in anti-obesity drug development: The critical role of hunger-promoting neurons. Nat. Rev. Drug Discov..

[28] Nijveldt R.J., van Nood E., van Hoorn D.E., Boelens P.G., van Norren K., van Leeuwen P.A. (2001). Flavonoids: A review of probable mechanisms of action and potential applications. Am. J. Clin. Nutr..

[29] Nakachi Y., Yagi K., Nikaido I., Bono H., Tonouchi M., Schonbach C., Okazaki Y. (2008). Identification of novel PPARgamma target genes by integrated analysis of ChIP-on-chip and microarray expression data during adipocyte differentiation. Biochem. Biophys. Res. Commun..

[30] Harris C.A., Haas J.T., Streeper R.S., Stone S.J., Kumari M., Yang K., Han X., Brownell N., Gross R.W., Zechner R. (2011). DGAT enzymes are required for triacylglycerol synthesis and lipid droplets in adipocytes. J. Lipid Res..

[31] Yen C.L., Stone S.J., Koliwad S., Harris C., Farese R.V. (2008). Thematic review series: Glycerolipids. DGAT enzymes and triacylglycerol biosynthesis. J. Lipid Res..

[32] Cao J., Li J.L., Li D., Tobin J.F., Gimeno R.E. (2006). Molecular identification of microsomal acyl-CoA:glycerol-3-phosphate acyltransferase, a key enzyme in de novo triacylglycerol synthesis. Proc. Natl. Acad. Sci. USA.

[33] Clarke S.L., Robinson C.E., Gimble J.M. (1997). CAAT/enhancer binding proteins directly modulate transcription from the peroxisome proliferator-activated receptor gamma 2 promoter. Biochem. Biophys. Res. Commun..

[34] Darlington G.J., Ross S.E., MacDougald O.A. (1998). The role of C/EBP genes in adipocyte differentiation. J. Biol. Chem..

[35] Hishida T., Nishizuka M., Osada S., Imagawa M. (2009). The role of C/EBPδ in the early stages of adipogenesis. Biochimie.

[36] Lu K., Han M., Ting H.L., Liu Z., Zhang D. (2013). Scutellarin from Scutellaria baicalensis suppresses adipogenesis by upregulating PPARα in 3T3-L1 cells. J. Nat. Prod..

[37] Mora S., Pessin J.E. (2002). An adipocentric view of signaling and intracellular trafficking. Diabetes Metab. Res. Rev..

[38] Bhattacharya I., Ullrich A. (2006). Endothelin-1 inhibits adipogenesis: Role of phosphorylation of Akt and ERK1/2. FEBS Lett..

[39] Manning B.D., Cantley L.C. (2007). AKT/PKB signaling: Navigating downstream. Cell.

[40] Lam E.W., Brosens J.J., Gomes A.R., Koo C.Y. (2013). Forkhead box proteins: Tuning forks for transcriptional harmony. Nat. Rev. Cancer.

[41] Nakae J., Barr V., Accili D. (2000). Differential regulation of gene expression by insulin and IGF-1 receptors correlates with phosphorylation of a single amino acid residue in the forkhead transcription factor FKHR. EMBO J..

[42] Nakae J., Kitamura T., Kitamura Y., Biggs W.H., Arden K.C., Accili D. (2003). The forkhead transcription factor Foxo1 regulates adipocyte differentiation. Dev. Cell.

